# Altered Gene Expression in Early Atherosclerosis Is Blocked by Low Level Apolipoprotein E

**DOI:** 10.1371/journal.pone.0002503

**Published:** 2008-06-18

**Authors:** Yanqing Ma, Craig C. Malbon, David L. Williams, Fayanne E. Thorngate

**Affiliations:** Department of Pharmacological Sciences, School of Medicine, Stony Brook University, Stony Brook, New York, United States of America; University of Sheffield, United Kingdom

## Abstract

**Background:**

Mice deficient in apolipoprotein E (apoE^−/−^) develop atherosclerosis. The possible linkage between expression of adhesion molecules/cofactors and atherosclerosis was probed at the level of mRNA and protein expression. The hypothesis of a linkage between changes of adhesion molecules/cofactors and atherosclerosis was tested further by suppression of aortic lesion formation in apoE^−/−^ mice by expression of very low levels of transgenic apolipoprotein E.

**Methodology/Principal Findings:**

We show that at 8.5 months of age, the apoE^−/−^ mice display elevated expression of mRNA for LFA-1, MAC-1, VCAM-1, ICAM-1, and for CD44, as well as MCP-1, cathepsin B, and COX-2 (but not that for eNOS) in atherosclerotic aortic arches. At earlier age, (10–13 week old) apoE^−/−^ mice already display elevated expression of mRNA of CD44, LFA-1, MAC-1, VCAM-1, ICAM-1, cathepsin, and of COX-2 in lesioned aortic arches. Expressing very low levels of transgenic apolipoprotein E suppresses both aortic lesions and the expression of mRNA of LFA-1, VCAM-1, MCP-1, cathepsin B, and of ICAM-1 in ApoE^−/−^ mice. We tested at the level of protein, the observations obtained for mRNA expression. CD11a (a component of LFA-1), VCAM-1 and cathepsin B expression was found to be elevated in apoE^−/−^ aortas at 8–9 months; low level expression of transgenic apolipoprotein E rectifies these changes.

**Conclusions/Significance:**

Atherosclerotic lesions in apoE^−/−^ mice are detected as early as 4 weeks of age. Expression of low levels of apoE is shown to be both atheroprotective and to suppress these changes in key adhesion and inflammatory molecules observed in early atherosclerotic lesions.

## Introduction

Atherosclerosis is a major contributor to the development of cardiovascular disease, a leading cause of morbidity and mortality in developed countries. Risk factors such as hypertension and hyperlipidemia account for ∼50% of the genetic risk for coronary heart disease [1]. Therapeutics more effectively targeted to key events in atherosclerosis are required to reduce its risk. Details of the underlying molecular basis for atherosclerosis remain obscure. Some important leads have been revealed through studies of knockout mice. Development of atherosclerosis (induced by a variety of means) has been shown to be attenuated in mice made deficient in adhesion molecules like P- and E-selectins or deficient in the chemokine receptor CCR2 [2]–[5]. These studies reinforce the notion that developing atherosclerosis involves an inflammatory response.

Mice-deficient in apolipoprotein E (apoE^−/−^) spontaneously develop pronounced aortic lesions characteristic of atherosclerosis [6]–[8]. Earlier studies of expression of genes in apoE^−/−^ mice on a fat-enriched “Western” diet [9]–[11] or stimulated with lipopolysachharide [12] focus essentially on analysis of atherosclerotic lesion formation at late stages. One of the earliest steps in lesion formation is the recruitment of monocyte/macrophages to the subendothelial space of the vessel wall. Our overarching goal was to investigate changes in key adhesion [*e.g.*, VCAM-1, ICAM-1, leukocyte ICAM-1 ligands CD11a/CD18 (LFA-1) and CD11b/CD18 (Mac-1) [13], and adhesion protein CD44 [14]] as well as chemokine [*e.g.*, MCP-1 [15]] molecules/cofactors [3] involved in monocyte adhesion during the initiation / early development of atherosclerotic lesions. Expression of macrophage-derived protein cathepsin B, the proatherogenic and antiatherogenic agent COX-2 [16], and the regulator of vascular tone eNOS, also would be of interest in analysis of developing atherosclerotic lesions. In the current work, gene expression of key inflammatory and macrophage molecules is investigated in apoE^−/−^ mice during the initiation and progression of early atherosclerosis, as well as in response to transgene expression of very low levels of apoE (<2 µg/ml) which are remarkably atheroprotective in apoE^−/−^ mice [17].

## Results

### mRNA expression of adhesion and inflammatory molecules in aortic arches of older apoE^−/−^ mice

**Figure 1:**
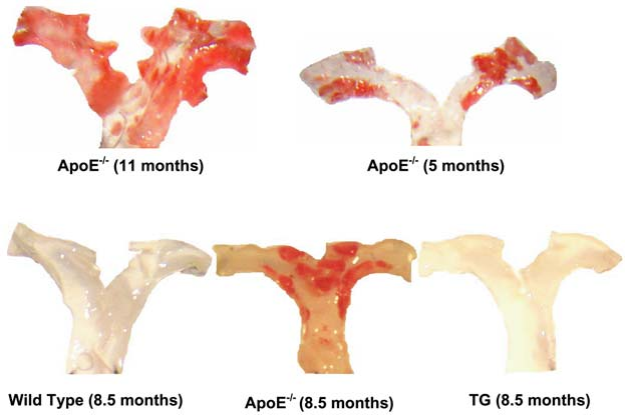
Aortic arch lesion development in older apoE-deficient (apoE^−/−^) mice. Mice were maintained on a chow diet. At 11-, 8.5- and 5-months of age: effects of expression of very low levels of apoE on atherosclerotic lesion development. Aortas were stained with Sudan IV to show neutral lipid deposits.

**Figure 2:**
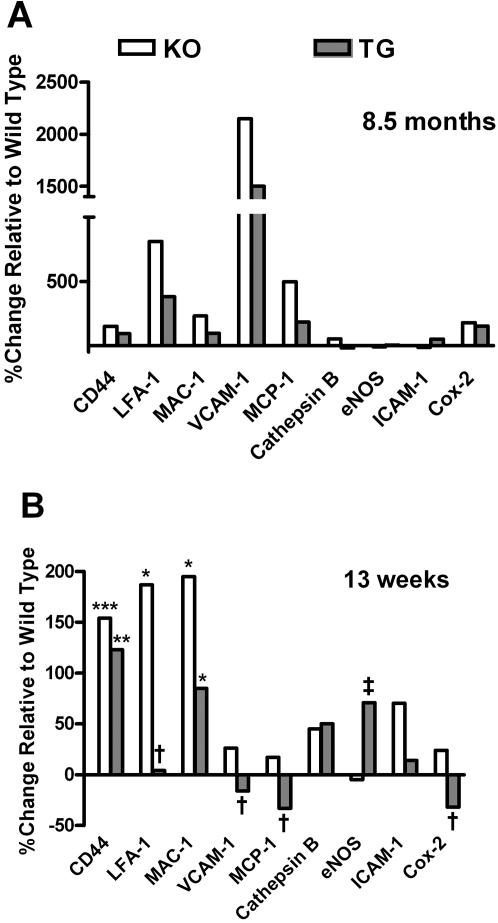
Gene expression of CD44, LFA-1, MAC-1, VCAM-1, MCP-1, cathepsin B, eNOS, ICAM-1, and COX-2 in of older mice. A, gene expression in pooled aortic arches expressed as % change relative to wild type (KO: *n* = 10; TG: *n* = 10; WT: *n* = 5) from 8.5 mo old mice on chow diet, all normalized to cyclophilin D mRNA levels. TG is apoE^−/−^ expressing low-level transgenic apoE, KO is apoE knockout, and WT is wild type. B, mRNA expression of adhesion molecules/cofactors in individual aortic arches (KO: *n* = 13; TG: *n* = 19, WT: *n* = 10) from 13 week old mice on a chow diet, normalized to cyclophilin D mRNA levels. CD44, LFA-1 and MAC-1 RNA levels were elevated in apoE^−/−^ mice compared to wild type mice (Bonferroni test, *p<0.05, **p<0.01, ***p<0.0001). Low-level apoE suppressed LFA-1, VCAM-1, MCP-1 and COX-2 gene expression in transgenic mice compared to apoE^−/−^ mice (Bonferroni test, †p<0.05, ‡p<0.01).

Mice made deficient in apoE (apoE^−/−^, KO) were maintained on a standard chow diet. In the absence of a fat-enriched diet or other stimulus, apoE^−/−^ display aortic arches rich in atherosclerotic lesions at 11- , 8.5- or 5- months of age, as made visible by staining of neutral lipids with Sudan IV stain (fig. 1). As others and we have previously observed, there is a fairly wide variation in the amount of atherosclerosis detected in apoE^−/−^ mice. There is very little variation in the transgenic mice, as there is almost total suppression of lesion formation [17]. Control mice displayed no such aortic lesions at either 8.5-months (fig. 1), 11- or 5-months of age (not shown). We first probed gene expression in the apoE^−/−^ mice that were 8.5 months of age, to optimize the detection of possible changes in mRNA levels of targeted gene products in the apoE^−/−^ mice. To accomplish this goal, aortic arches were pooled from either 8.5-months old apoE^−/−^ or from control wild type mice; mRNA was analyzed by real time PCR amplification. At 8.5-months of age, mRNA levels of adhesion molecules CD44 and VCAM-1, their ligands LFA-1and MAC-1, and the chemoattractant MCP-1 were elevated by apoE deficiency (KO) as compared to mRNA levels of aortic arches obtained from wild type (WT) mice (fig. 2A). The increases ranged from 22.5-fold (VCAM-1), 9.1-fold (LFA-1), 6.0-fold (MCP-1), 2.8-fold (COX-2) to 2.5-fold (CD44), with lesser increases in cathepsin-B and Cox-2, and no change from WT for mRNA levels of eNOS (fig. 2A).

For apoE^−/−^ mice engineered with a transgene to express very low (<2 µg/ml) circulating levels of adrenal apoE (TG) that are atheroprotective, atherosclerotic lesion formation is essentially absent at 8.5 months (fig. 1). We next compared mRNA expression of key adhesion molecules in aortic arches prepared from these mice with those from apoE^−/−^ mice (fig. 2A). The mRNA values for the adhesion molecules/cofactors that were elevated in the apoE^−/−^ mice were reduced in their transgenic counterparts that express very low levels of adrenal apoE. mRNA levels for CD44 declined from 2.5- to 1.9-fold, for VCAM-1 from 22.5- to 16.0-fold, for LFA-1 from 9.1- to 4.8-fold, and for MCP-1 from 6.0- to 2.8-fold (fig. 2A). Expression of low levels of apoE suppresses the expression of mRNA of these key adhesion molecules in aortic arches and was atheroprotective. This suppression occurred even against a background of high plasma cholesterol (transgenic: 434.7±55.1 mg/dl, knockout: 380.5±70.6 mg/dl, p = 0.55) which is equivalent between the two groups. Protection from formation of atherosclerotic lesions in apoE^−/−^ mice by very low levels of apoE occurs, although the suppression of the mRNA expression of the key molecules did not reach wild type levels. Cathepsin-B gene expression in apoE^−/−^ KO mice, for example, was higher than in wild-type mice (∼1.5 fold). Expression of the apoE transgene, however, yields only a modest decline in cathepsin-B mRNA when compared to that of the WT mice. ICAM-1 and eNOS gene expression was found to display only minor differences among all three groups (KO, TG, and WT, fig. 2A).

### mRNA expression in aortic arches of 13-weeks old mice

A primary goal of this work was to extend the analysis of mRNA to mice in earlier stages of lesion formation, recognizing that the amounts of starting tissue become a technical challenge. To this end, we refined methods to measure mRNAs in aortic arches surgically removed from individual mice, rather than in preparations from pooled aortic arches (fig. 2A). We exploited the technology and pressed the analysis of lesion formation in progressively younger mice (fig. 2B). At 13 weeks of age, apoE^−/−^ mice displayed substantially elevated levels of mRNA for adhesion proteins CD44 (2.5-fold), ICAM-1 (1.7-fold), the adhesion ligand LFA-1 (2.9-fold), and MAC-1 (3.0-fold) in their aortic arches. Each of the other adhesion molecules/cofactors displayed mRNA levels in apoE^−/−^ mice that were only slightly higher or equivalent to those prepared from aortic arches of the WT mice.

The expression by transgene (TG) of very low levels of adrenal-derived apoE in the 13-week old apoE^−/−^ mice again resulted in sharp reductions in the elevated mRNA levels of LFA-1, MAC-1, ICAM-1 MCP-1, VCAM-1 and Cox-2 (fig. 2B). Of interest, mRNA levels for VCAM-1, MCP-1, and Cox-2 in aortic arches of apoE^−/−^ mice were suppressed by transgenic expression of very low apoE, reaching levels well below those observed in WT mice (fig. 2B). Expression of CD44 mRNA and cathespsin-B mRNA, in contrast, was largely unaffected by the transgenic expression of low levels of apoE in the apoE^−/−^ mice. mRNA levels of eNOS in the apoE^−/−^ mice harboring the transgene (TG) were higher than either apoE^−/−^ (KO) mice (*p*<0.01) or WT mice (*p*<0.05). Again, as with the 8.5 month mice, the suppression is observed in the face of elevated plasma cholesterol (transgenic: 352.8±41.7 mg/dl, knockout: 266.4±46.9 mg/dl, p = 0.29) as compared to the wild type, 119.0 mg/dl±27.6.

**Figure 3:**
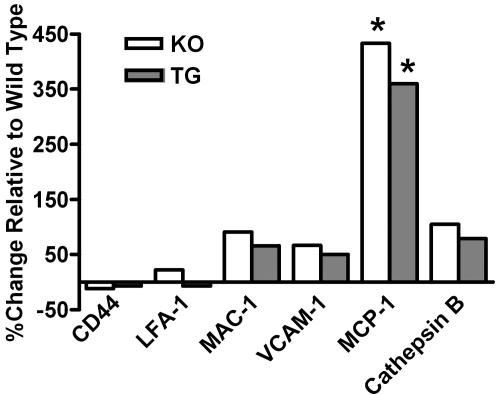
CD44, LFA-1, MAC-1, VCAM-1, MCP-1 and cathepsin B mRNA levels in 13 week old mouse blood leukocytes. Gene expression in blood leukocytes from 13 week old mice on a chow diet, all normalized to cyclophilin D and expressed as % change relative to wild type (KO: *n* = 22; TG: *n* = 12; WT: *n* = 11). TG is apoE^−/−^ expressing low-level transgenic apoE, KO is apoE knockout, and WT is wild type. Leukocytes were prepared as described in *Materials and Methods*. The gene expression levels were not different between the groups for all the genes tested except for MCP1, which was significantly elevated in the KO and TG groups (Bonferroni test, *p<0.0001) relative to wild type.

Expression of mRNAs for these key molecules also was examined in peripheral blood leukocytes. mRNA levels of the adhesion molecules/cofactors were investigated in peripheral leukocyte preparations to ascertain if mRNA levels in these circulating cells would mirror those observed in the aortic arches. Unlike the aortic arch preparations, the mRNA levels for these molecules largely did not differ among the WT, KO, and TG genotypes in preparations obtained from peripheral blood leukocytes. Only the mRNA levels of MCP-1 were found to be significantly elevated in peripheral blood leukocytes from the 13-week old apoE^−/−^ mice (fig. 3). This elevated MCP-1 mRNA level remained elevated in the leukocytes of apoE^−/−^ mice harboring the transgene (fig. 3).

### mRNA expression in aortic arches of 10-weeks old mice

**Figure 4:**
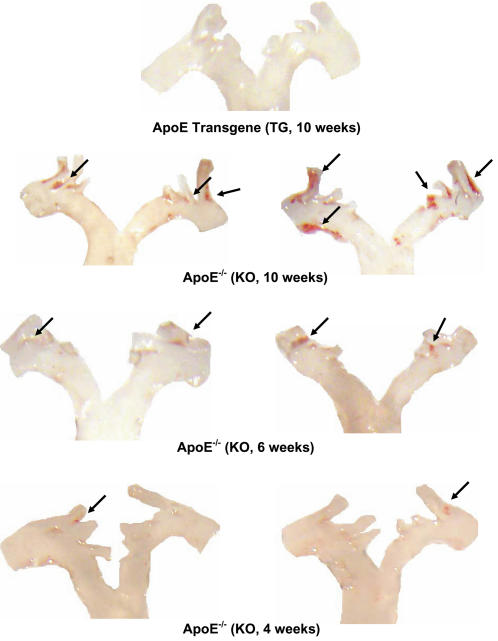
Aortic arch lesion development in apoE^−/−^ : effects of expression of very low levels of apoE. Aortas were stained with Sudan IV to show deposits of neutral lipid. Atherosclerotic lesions appeared in the aortic arch of apoE^−/−^ as early as 4 weeks old. More lesions were observed as the mice aged. For apoE^−/−^ mice expressing very low levels of apoE, no aortic arch lesions were detected. The arrows point to lesions.

**Figure 5:**
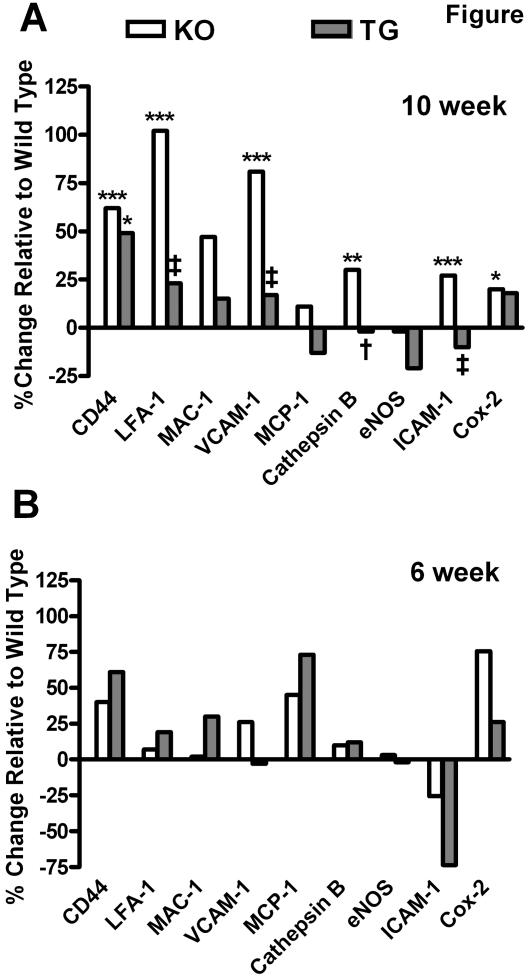
Gene expression of CD44, LFA-1, MAC-1, VCAM-1, MCP-1, cathepsin B, eNOS, ICAM-1, and COX-2 in younger mice. Gene expression of CD44, LFA-1, MAC-1, VCAM-1, MCP-1, cathepsin B, eNOS, ICAM-1, and COX-2 in individual aortic arches expressed as % change relative to wild-type (*n* = 8 for all groups) from 10- (A) week old mice. Data from 6- (B) week old mice are from pooled aortas, due to the small sample size. Both sets of mice were on chow diet and normalized to cyclophilin D mRNA levels. ApoE^−/−^ mice had higher CD44, LFA-1, VCAM-1, cathepsin B, ICAM-1 and COX-2 gene expression than wild type mice (Bonferroni test, *p<0.05, **p<0.01, ***p<0.0001). Low-level apoE in transgenic mice suppressed the gene expression of LFA-1, VCAM-1, cathepsin B and ICAM-1 compared to apoE^−/−^ mice (Bonferroni test, †p<0.05, ‡p<0.0001).

We investigated the presence of atherosclerotic lesions in younger apoE^−/−^ mice in an effort to probe changes in the mRNA of key molecules in lesions of involved aortic arches (fig. 4). Lesion formation was obvious in arches of the 10-weeks old apoE^−/−^ mice (fig. 4). The presence of the adrenal apoE transgene again effectively suppresses the formation of the lesions in aortic arches of 10-week old (fig. 4) and younger (data not shown) apoE^−/−^ mice. We analyzed the mRNA expression in these preparations cognizant that the ratio of lesion to non-lesioned tissue in the arches was declining in younger animals and “diluting out” any changes that may exist. Using individual aortic arches of 10-week old apoE^−/−^ mice we were still able to detect increased expression of mRNA of CD44, LFA-1, VCAM-1, ICAM-1, COX-2, and cathepsin B in comparison to mRNA expression in aortic arches of the WT mice (fig. 5A). As observed in the 8.5-month and 13-week apoE^−/−^ mice, the enhanced expression of LFA-1, ICAM-1, and VCAM-1 mRNA were sensitive to the expression of the very low levels of adrenal apoE produced in the apoE^−/−^ mice harboring the transgene (fig. 5A). Cathepsin-B mRNA expression also was significantly suppressed in aortic arches of 10-week old apoE^−/−^ mice harboring the transgene. mRNA levels of MCP-1 and eNOS in aortic arch preparations among the three 10-week-old groups (KO, TG, WT), in contrast, did not vary significantly. As for the previous age groups, there is no difference between the plasma cholesterol levels in the two knockout groups, which are both elevated (transgenic: 304.9±82.6, knockout: 307.9±70.0, p = 0.98).

### mRNA expression in aortic arches of 6-weeks old mice

Atherosclerotic lesions were detected at 6-weeks and even at 4-weeks of age in the apoE^−/−^ mice (fig. 4). We probed the mRNA levels for these same molecules, key to inflammation and macrophage activation, in aortic preparations prepared from mice at 6 weeks of age (fig. 5B). Severely limited by the amount of starting tissue, the analysis of mRNA in aortic arches of 6-week-old mice required that we pool aortic arches from at least 6 animals to obtain reliable measurements. The mean levels of mRNA for CD44, VCAM-1, MCP-1, and Cox-2, in fact, were elevated in 6-weeks old apoE^−/−^ mice (fig. 5B). The trend of the transgene expression of very low levels of apoE suppressing VCAM-1, ICAM-1 and Cox-2 mRNA expression observed in the 8.5-months old and 10–13 weeks old apoE^−/−^ mice appears to extend to the pooled aortic preparations from the 6-weeks old mice, although the changes were less marked. The technical difficulties encountered with analysis of small lesions on very small aortic aches of 6-weeks old mice precluded analysis at the mRNA level of tissue from aortic arches of 4-weeks old apoE^−/−^ mice, although the arches do display some very early atherosclerotic lesions (fig. 4).

### Expression of adhesion and inflammatory molecules at the protein level in lesioned and atheroprotected aortas of apoE^−/−^ mice

**Figure 6:**
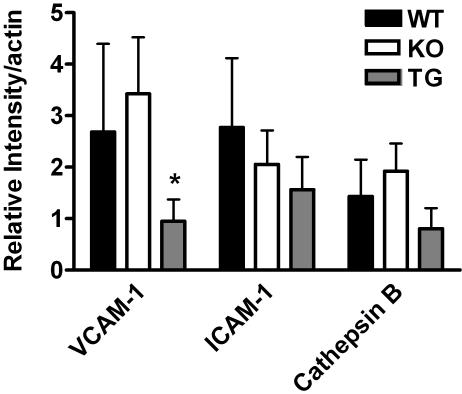
Protein Levels of VCAM-1 and cathepsin B in aortas. Total aorta protein from apoE^−/−^ chow-fed mice at 8–9 months of age (100 µg protein/lane) was resolved by 10% denaturing SDS-PAGE and Western blots conducted as described in the *Materials and Methods*. Six separate aorta lysates were run each for wild type (WT), apoE^−/−^ (KO), or apoE^−/−^ expressing low-level transgenic apoE (TG ). The image bands were scanned and analyzed using Image Quant software. Actin was immunostained as a loading control (Mann Whitney test, *p<0.03).

We investigated the changes observed in mRNA for VCAM-1 and cathepsin B at the protein level in aortas from 8–9 month-old mice. The expression of wild type (WT), apoE^−/−^ (KO), and apoE^−/−^ mice+transgenic mice (TG, fig. 6), as determined by immunoblotting of samples (0.1 mg protein) of aorta lysates subjected to SDS-PAGE and electrophoretic transfer of resolved proteins to blots. There was a trend toward increased expression of VCAM-1 and cathepsin-B in aorta lysates of the apoE^−/−^ mice. A wide variation in the level of lesion burden in apoE^−/−^ mice complicates an immunoblot analysis. At the same age, apoE^−/−^ mice can display extensive lesion development or little [17]. Expression of the very low levels of apoE from the adrenal, reversed the increase in expression provoked in the apoE^−/−^ mice. The presence of the transgene is sufficient to suppress the protein expression of VCAM-1 relative to WT and apoE^−/−^ (*p*<0.05, *n* = 6). Actin levels were measured in the immunoblots to verify equivalent protein loading.

### In situ staining of LFA-1 and VCAM-1expression in WT, KO, and KO+TG mice

**Figure 7:**
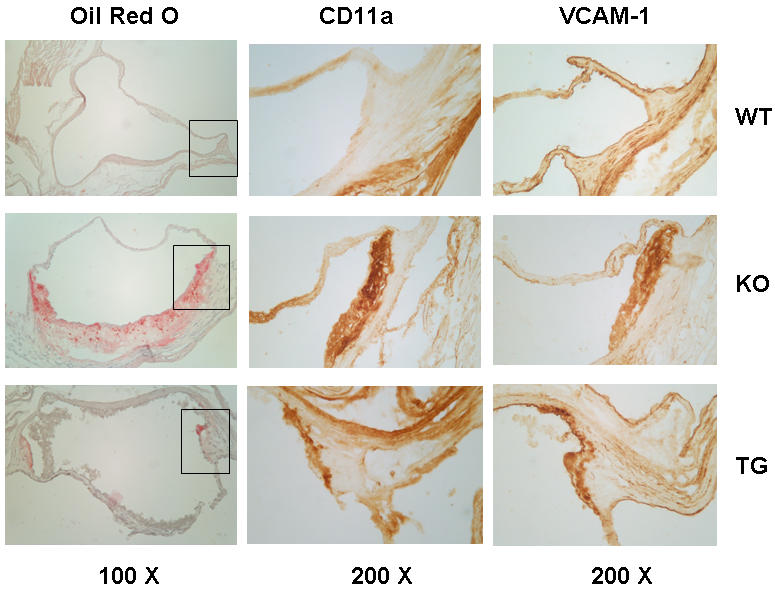
Immunohistochemistry of aortic root lesions. Aortas from 8–9 month chow fed male mice were fixed and sectioned as described in *Materials and Methods*. Sections were stained for neutral lipid accumulation with oil red O, then adjacent sections were immunostained for VCAM-1 and CD11a (LFA-1) as described. Wild type (WT), apoE^−/−^ (KO), and apoE^−/−^ expressing low level transgenic apoE (TG) are indicated for each row. Representative photomicrographs are shown, with the magnifications at the bottom of each column. The areas in the boxes in the oil red O column are magnified in the VCAM-1 and CD 11a columns.

Our mRNA analysis enabled us to detect elevated expression of key molecules in the aortas of mice with atherosclerotic lesions (apoE^−/−^), advancing from detection in 8.5 months to 6–10 weeks of age. Further, our ability to make use of the adrenal transgene to express very low levels of apoE sufficient to suppression lesion formation in apoE^−/−^ mice was key in testing linkages between the changes in mRNA and the lesion formation. In the cases of over expression of cathepsin-B and VCAM-1 in apoE^−/−^ mice, analysis by immunoblotting was able to confirm the results obtained at the level of message. To advance the analysis further, we probed *in situ* immunohistochemically aortic root areas from 8–9 month mice from the WT, KO, and KO+TG groups (fig. 7). Oil red O staining was employed to locate forming lesions (*i.e.*, regions of lipid deposition). Adjacent sections of these aortic preparations were then stained for VCAM-1 and LFA-1 (the CD11a component of LFA-1). Unlike VCAM-1 antibodies, antibodies available against CD11a were found to be of little use in immunoblotting assays.

Oil red O staining for neutral lipids was absent in the aortic root areas of WT mice, extensive in the apoE^−/−^ KO mice, and much less detectable in the KO+TG mice (left-handed panel, fig. 7). Areas of oil red O staining enclosed by the boxes are magnified in the center (CD11a staining) and right-handed (VCAM-1 staining) panels. The adhesion marker staining closely followed the lipid staining in the lesion areas, with little to none seen in the WT aortic root, even in the lesion-prone shoulder areas of the valves. In the equivalent area of the apoE^−/−^ aorta there was abundant staining for both CD11a (center panel) and VCAM-1 in cells that appear to be lipid loaded macrophages (left-handed panel, fig. 7). In the aortic roots of the KO+TG mice there was only a small amount of staining for both adhesion molecules. This limited staining in the KO+TG mice tissue was localized to an area with some residual lipid deposition. The amount of staining of adhesion molecules in apoE^−/−^ mice harboring the apoE adrenal transgene was considerably less than that observed in the apoE^−/−^ mice (fig. 7).

## Discussion

Recruitment of circulating monocytes to the arterial *intima* is an early event in formation of atherosclerotic lesions [18]. Adhesion molecules and cofactors such as cytokines, which can tether and activate integrin complexes, initiate the movement of monocytes and T-lymphocytes from the circulation into the vessel wall [19]. Changes in the expression of adhesion molecules and their cofactors might be critical to initiation and/or progression of atherosclerotic lesions. We tested this hypothesis directly through analysis of key adhesion and inflammatory molecules in atherosclerotic lesions of aortic arches of apoE^−/−^ mice. The leukocyte ligands LFA-1 and MAC-1 for the adhesion molecule ICAM-1 have been implicated in lesion formation [11], [20]. Increased expression of LFA-1 and MAC-1 mRNAs was observed in lesioned aortic arches of apoE^−/−^ mice at 8.5 months as well as 13-weeks of age. Up-regulation of LFA-1 mRNA in aortic arches of apoE^−/−^ mice was detected as early as 10 weeks (2-fold), progressing in 13-weeks (2.9-fold), and 8.5-months (9.1-fold) old KO mice. mRNA levels for MAC-1 increased in the aortic arches of apoE^−/−^ mice, increasing 3.0-fold at 13-weeks of age and remaining at 3.3-fold at 8.5-months of age. Up-regulation of ICAM-1 adhesion molecule was detected in apoE^−/−^ mice, as early as 10- and 13-weeks of age. By 8.5 months of age, ICAM mRNA returned to normal levels, suggesting that ICAM, LFA-1, and MAC-1 may play more important roles in the initiation and/or early progression of atherosclerotic lesions.

VCAM-1 is an immunoglobulin-like adhesion molecule expressed on activated endothelial cells. VCAM-1 promotes monocyte adhesion and accumulation on the vessel wall at lesion-prone sites [21]. VCAM-1 mRNA levels increased by more than 20-fold at 8.5-months of age. Increased expression of adhesion molecule VCAM-1 mRNA was observed in aortic arches of apoE^−/−^ mice as early as 10- and 13- weeks of age. Immunoblotting data and immunohistochemical analysis of VCAM-1 expression support a role of this adhesion molecule in formation of atherosclerotic lesions. Deficiency of VCAM-1 has been shown to diminish the formation of early foam cell lesions throughout the aorta of low-density lipoprotein receptor-deficient (LDLR^−/−^) mice, promoting VCAM-1 as a major role in the initiation of atherosclerosis [22]. In good agreement, we found that suppression of lesion formation in apoE^−/−^ mice with very low levels of apoE, levels not capable of rectifying the elevated lipids observed in apoE^−/−^ mice, were accompanied by a sharp decline in VCAM-1 mRNA and protein expression.

The chemoattractant cytokine MCP-1 is well known to stimulate macrophage recruitment in atherosclerosis, and so it was a high value target for our studies. MCP-1 gene expression, as measured by mRNA levels, in aorta was increased by 6.0 fold in apoE^−/−^ mice at 8.5-months of age. Furthermore, expression of MCP-1 mRNA was found to be elevated even earlier, at 10- and 13-weeks of age in apoE^−/−^ mice. Since levels of expression of MCP-1 mRNA appeared to increase with the age of the apoE^−/−^ mice, MCP-1 may be playing a more important role in the later rather than early stages of atherosclerosis. Expression of low levels of apoE from the transgene suppressed aortic lesions and attenuated the increase in MCP-1 in apoE^−/−^ mice. In mice lacking either MCP-1 [15] or its receptor, CCR2 [3] atherosclerosis is depressed. The pattern of changes of MCP-1 gene expression was similar to that of the VCAM-1 gene. It should be noted that both MCP-1 and VCAM-1 genes display NF-κB consensus DNA-binding sites in the 5′- flanking regions [23], [24]. In contrast, the circulating leukocyte levels of MCP-1 were elevated in both the apoE^−/−^ and transgenic mice, perhaps indicating different roles for blood leukocyte and vessel wall expressed MCP-1 in the low level apoE suppression of atherosclerosis.

The hyaluronan receptor CD44 plays a critical role in the progression of atherosclerosis through multiple mechanisms, including inflammatory cell recruitment and cellular activation [14], [23], [25]. CD44 mRNA levels in aorta of the apoE^−/−^ mice were increased at 10-weeks of age (1.6-fold), at 13-weeks of age (2.5-fold), maintaining elevated levels at 8.5 months of age (2.4-fold). The expression of low levels of apoE, however, only had a modest effect on CD44 mRNA levels in aortic arches of apoE^−/−^ mice, suggesting a less obvious linkage of CD44 and the atheroprotective effects of apoE.

Finally, cathepsins are macrophage lysosomal cysteine proteases that can accelerate the degradation of extracellular matrix at sites of inflammation, which may include cathepsin B [26]. Our results showing increased expression of cathepsin B mRNA in the aorta of apoE^−/−^ mice, as early as 10 weeks of age, support this possible role. Furthermore, elevated cathepsin B mRNA levels were suppressed in the apoE^−/−^ mice expressing atheroprotective levels of apoE by transgene. Increased expression of cathepsin B gene can be detected in apoE^−/−^ mice following a 20-week diet high in fat content [9]. Cathepsin B has the ability to degrade native and modified low-density lipoprotein, which in turn can provoke accumulation of modified LDL in the vessel wall and promote foam cell formation [27]. In humans, atherosclerotic aortas also show increased cathepsin activity [28].

The suppression of these messages occurred even with elevated plasma cholesterol in both the apoE^−/−^ and the low level apoE mice. The total plasma cholesterols for both of these groups were not significantly different at any age. We previously showed that the lipoprotein particle distributions were identical between these groups of mice by FPLC lipoprotein profile analysis, and the highly sensitive technique of laser light scattering[17]. This makes it unlikely that the effects we observed on vessel wall mRNAs is due to apoE's well-characterized role in lipoprotein clearance. Previous work has shown that apoE blocks mitogen-stimulated T-lymphocyte activation[29]–[31], an effect that might restrict inflammatory responses in atherosclerotic lesions. In cell culture studies, apoE was found to inhibit platelet aggregation[32], to inhibit platelet-derived growth factor (PDGF)-stimulated migration and proliferation of vascular smooth muscle cells[33] , and to suppress TNF α-stimulated VCAM-1 expression in endothelial cells[34] actions that could be anti-atherogenic within vascular lesions. Cell culture studies suggest that apoE disrupts PDGF signaling in smooth muscle cells via interaction with LRP[35], [36] . We are currently studying the receptors and signaling pathways that may be involved in mediating the actions of low level apoE we observed in these mice.

Remarkably, atherosclerotic lesion formation was detectable as early as 4-weeks of age in apoE^−/−^ mice, an age at which details about atherogenesis are few. By profiling of mRNA expression of key molecules involved in inflammation and activation of macrophages we succeeded in extending the characterization of mRNA expression of many of these molecules in apoE^−/−^ mice to as early as 10-weeks of age. The availability of apoE^−/−^ mice in which low levels of apoE expression by an adrenal transgene is atheroprotective enabled us to test the changes in mRNA with the onset of the lesions as well as with the atheroprotective properties of low-level apoE secretion. Relatively small, diffuse lesions in aorta of 4- to 6-week old apoE^−/−^ mice, made visible by oil red O staining of lipid, now focus our attention on the initiation and early development of the atherosclerotic lesions. In future work we will employ techniques in which analysis of such small lesions will not be “diluted” in biopsies by the presence of adjacent, largely healthy aortic tissue.

## Materials and Methods

### Animals

Mice were maintained in a specific pathogen-free environment on a 12 h light, 12 h dark cycle. Only males were recruited into this study and the mice were on a chow diet containing 4.5% fat (PicoLab Rodent Diet 20, LabDiet) and water *ad libitum*. Wild type, apoE deficient (apoE^−/−^) mice, and the 619 transgenic line, which expresses too little apoE (<1% to 2% of wild type) to correct their plasma cholesterol levels [17] were on a mixed 9∶1 FVB/N×C57BL/6 genetic background. Transgenic mice and apoE^−/−^ mice used were siblings. Four groups of mice at different ages were studied for RNA expression analysis: in the first group (8.5±0.4 month), aortic arches of 10 apoE^−/−^ mice, 10 apoE^−/−^ with low transgenic apoE and five wild type mice were pooled. In the second group (12.9±1.4 week), aortic arches (apoE^−/−^: *n* = 13, apoE^−/−^ with low transgene: *n* = 19, wild type: *n* = 10) were collected individually. In the third group (10.1±0.2 week), eight aortic arches of apoE^−/−^, apoE^−/−^ with transgene and wild type mice were collected individually. In the fourth group (5.9±0.1 week), aortic arches of six each apoE^−/−^, apoE^−/−^ with transgene and wild type mice were pooled. Mice were sacrificed by exsanguination under Ketamine Hydrochloride (100 mg/ml, Abbott Laboratories) and Xylazine (20 mg/ml, Phoenix Scientific Inc.) (3∶2) anesthesia. The Stony Brook University Committee on Laboratory Animal Resources approved housing and experimental procedures.

### RNA extraction

Aortas were perfused with RNA*later* (Ambion) by heart puncture, adventitia removed, and the arches stored in RNA*later* at −80°C prior to RNA extraction. In the younger groups, blood was collected by heart puncture before perfusion and total RNA was extracted from total circulating white cells remaining after red cell lysis. Aortic arches were homogenized in TRIZOL Reagent and total RNA was extracted according to the manufacturer's instructions (Invitrogen). RNA concentration was measured using RiboGreen™ RNA Quantitation Reagent, (Molecular Probes). The RNA yield from individual mice was 0.3 µg to 1.0 µg for the aortic arch and 2.7 to 4.2 µg for white cells. First strand cDNA was synthesized using 1^st^ Strand cDNA Synthesis Kit (Roche Diagnostic).

### Preparation of DNA standards

VCAM-1, ICAM-1, LFA-1, COX-2 and Cyclophilin D cDNA cloning was performed using a PCR-Script™ Amp cloning Kit (Stratagene) with cDNA sequences obtained from GenBank. MCP-1 (Image clone ID; 5351355), LFA-1 (Image clone ID: 4015574), MAC-1 (Image clone ID: 3810731) and Cathepsin B (Image clone ID: 1887225) cDNA clones were purchased from ATCC. eNOS and CD44 clones were kindly provided by Dr. Philip A. Marsden's laboratory at the University of Toronto and Dr. Thomas P. St. John (ICOS Corporation) respectively. All clones were confirmed by sequencing. Standard curves were built using serial dilutions of cDNA (1/10 dilution, 1–10^−6^ ng).

Real-time PCR-Primers were designed for each gene using Primer3 software (1997 Whitehead Institute for Biomedical Research) or GeneRunner (version 3.00, 1994 Hastings Software, Inc). Amplicons of 50–300 bp with Tm 57–63°C and G/C content 30–80% were selected. Cyclophilin D was used as an endogenous control. The sequences of primers and product lengths are as follows: LFA-1, ATGCACCAAGTACAAAGTCAGC and TTGGTCGAACTCAGGATTAGC (202 bp); MAC-1, CTGAACATCCCATGACCTTCC and GCCCAAGGACATATTCACAGC (209 bp); Cathepsin B, TGACCAGTGCCCATGACAAGC and CATTGTTCCCGTGCATCAAAGG (229 bp); CD44, TCCTTCGATGGACCGGTTACC and GTGGAGCCGCTGCTGACATC (137 bp); VCAM-1, TACCAGCTCCCAAAATCCTG and TCTGCTAATTCCAGCCTCGT (152 bp); ICAM-1, GTGATCCCTGGGCCTGGTG and GGAAACGAATACACGGTGATGG (278 bp); MCP-1, CTGGATCGGAACCAAATGAG and AAGGCATCACAGTCCGAGTC (203 bp); eNOS, TGACCAGCACATTTGGCAATGG and CATGAGCGCTGCTGCAAAGC (65 bp); COX-2, GCCCAGCACTTCACCCATCAG and AAGTCCACTCCATGGCCCAGTC (86 bp); Cyclophilin D, TCCAAGAACCCGCGAGTCTTC and CCGGTCGTGGACCCAGTT (152 bp).

Real-time PCR was carried out in a 20 µl volume with 1 µl reverse transcription reaction of plasmid DNA, 0.3 µM primer and 10 µl 2× QuantiTect SYBR Green PCR Master Mix (QIAGEN). PCR reactions were run in the LightCycler system (Roche Molecular Biochemicals) following manufacturer protocols. Each sample was run in duplicate and mean values were used for analysis. Sample concentration was determined from the standard curve for each gene. Quantification of target gene mRNA was expressed as the ratio of the target gene to cyclophilin D. Melting curve analysis was performed to differentiate specific product from non-specific PCR products or primer-dimers.

### Determinations of atherosclerosis

Mice at different mean ages (11 month, 8.5 month, 5 month, 10 week, 6 week and 4 week) were sacrificed. Aortas were perfused with PBS and dissected, then fixed in neutral buffered formalin for 48 hours at 4°C. Aortas were stained with Sudan IV, as previously described [37]. The aortic tree was opened longitudinally and lesion images were captured with a macro lens and digital camera.

### Western Blots

Lysates were made from aortas (arch and thoracic segments) from male chow-fed mice 8–9 months old, as described [17]. 100 µg of total lysate protein per lane was resolved by 10% denaturing SDS-PAGE. The resolved proteins were transferred electrophoretically to a nitrocellulose membrane and immunostained using a rabbit anti-actin antibody (SIGMA), affinity purified goat anti-mouse VCAM-1, or cathepsin B (R&D Systems). The secondary antibody was either a peroxidase-coupled anti-goat IgG (R&D Systems) or peroxidase-coupled anti-rabbit IgG (GE Healthcare Lifesciences). Bands were visualized by enhanced chemiluminescence (Pierce). The blot was stripped between each immunostaining using Restore™ Western Blot Stripping Buffer (Pierce). To quantitate the protein bands, films of the blots were scanned and the images analyzed with Image Quant 5.2 (Molecular Devices).

### Immunohistochemistry

Mice were perfused through the left ventricle with PBS followed by 4% formaldehyde in PBS. Frozen sections through the aortic root were stained with oil red O and lightly counterstained with hematoxylin as previously described [38]. The primary antibodies used for immunohistochemistry of adjacent frozen sections were the anti-VCAM-1 above, and a rat anti-mouse CD11a monoclonal antibody (eBioscience). A Vectastain ABC *Elite* kit (Vector Laboratories) was used for detection, with DAB staining, and hematoxylin counterstaining.

### Statistical analysis

In the 8.5-months and 6-weeks old groups, more than two-fold change in mRNA expression was considered significant enough to warrant further investigation. For the 13- and 10-weeks old groups, Statistics Package for the Social Sciences (SPSS version 10.0.1, 1999, SPSS Inc, Chicago, IL) was employed for data analysis. Analysis of variance was performed comparing the mRNA expression levels among the groups with Bonferroni *post hoc* tests. Non-parametric tests were employed to analyze the unevenly distributed data. The protein data were analyzed using unpaired t-tests. Values were expressed as mean±S.E.M., unless otherwise indicated. A value of *p*<0.05 was considered significant.
